# Supplementary material for: Selenium Alleviates Cadmium Toxicity by Regulating Carbon Metabolism, AsA-GSH Cycle, and Cadmium Transport in Glycyrrhiza uralensis Fisch. Seedlings
Source: Plants (Basel). 2025 Jun 6;14(12):1736. doi: 10.3390/plants14121736 (PMC12196552; doi:10.3390/plants14121736)
Supplement: Supplementary file 1 [file plants-14-01736-s001.zip › plants-3661821-supplementary.pdf]

Table. S1 Sequencing Quality Information

Sample	Raw Reads	Raw Bases	Clean Reads	Clean Bases	Error Rate	Q20	Q30	GC Content
CK1_12h	21587934	6.48G	21297955	6.39G	0.03%	97.93%	93.78%	45.35%
CK2_12h	21000612	6.30G	20816910	6.25G	0.03%	97.93%	93.86%	45.47%
CK3_12h	19839327	5.95G	19684407	5.91G	0.02%	98.33%	94.82%	45.42%
Cd1_12h	21733338	6.52G	21545850	6.46G	0.02%	98.19%	94.51%	45.43%
Cd2_12h	23216709	6.97G	23032232	6.91G	0.02%	98.26%	94.63%	45.43%
Cd3_12h	19980168	5.99G	19824428	5.95G	0.02%	98.48%	95.18%	45.42%
Cd+Se1_12h	21347064	6.40G	21166994	6.35G	0.02%	98.23%	94.55%	45.43%
Cd+Se2_12h	20859552	6.26G	20703258	6.21G	0.02%	98.42%	95.03%	45.35%
Cd+Se3_12h	23194186	6.96G	23012262	6.90G	0.02%	98.36%	94.90%	45.29%
CK1_30d	23972258	7.19G	23743029	7.12G	0.03%	97.83%	93.67%	45.59%
CK2_30d	20401000	6.12G	20237085	6.07G	0.02%	98.41%	95.04%	45.55%
CK3_30d	21902935	6.57G	21691900	6.51G	0.03%	97.85%	93.70%	45.61%
Cd1_30d	21908678	6.57G	21730542	6.52G	0.03%	97.95%	93.86%	45.47%
Cd2_30d	22291839	6.69G	22108771	6.63G	0.03%	98.10%	94.24%	45.47%
Cd3_30d	20970002	6.29G	20797780	6.24G	0.03%	98.03%	94.04%	45.42%
Cd+Se1_30d	21720302	6.52G	21538481	6.46G	0.03%	97.93%	93.83%	45.52%
Cd+Se2_30d	21839257	6.55G	21678617	6.50G	0.02%	98.51%	95.19%	45.40%
Cd+Se3_30d	19606754	5.88G	19461852	5.84G	0.02%	98.23%	94.48%	45.38%

Table. S2 The forward and reverse primer sequences used in qRT-PCR for verification.

Gene	Name	Forward primer（5'-3'）	Reverse primer（5'-3'）
Glyur002632s00037709	YSL1	ACACAGGGGAACAAAATGGC	AATCATCCCTGCTCCCACAA
Glyur001554s00031225	NRAM5	TGCTCTGCCGAAAACCTTTC	TGCATGATGTATTGCCCTGC
Glyur000191s00015621	IRT3	CACTCTCGCGCTCATTTCAA	GGGAAGGGTTGAGATTGGGA
Glyur000320s00014335	HMA5	GAGGTTACCGCCGTTTTCTC	CCAGCATCTTCAATGGCCTC
Glyur000439s00019016	HMA4	CGCGGACCATATTTCAAGGG	GAGCACATGGACAAGCAACA
Glyur000039s00004256	MTP11	TGCTGCCAAAGTTTATGCGT	ATTCCCAATGGTTGCATCCG
Glyur001025s00025595	ZIP6	GGGGAAGAGTTGGTTAGGCT	CCAAAGCTAAATCCCGCCTG
Reference genes	18S rRNA	TCCGGCGCTGTTACTTTGAA	ATCCCGAAGGCCAACACAAT

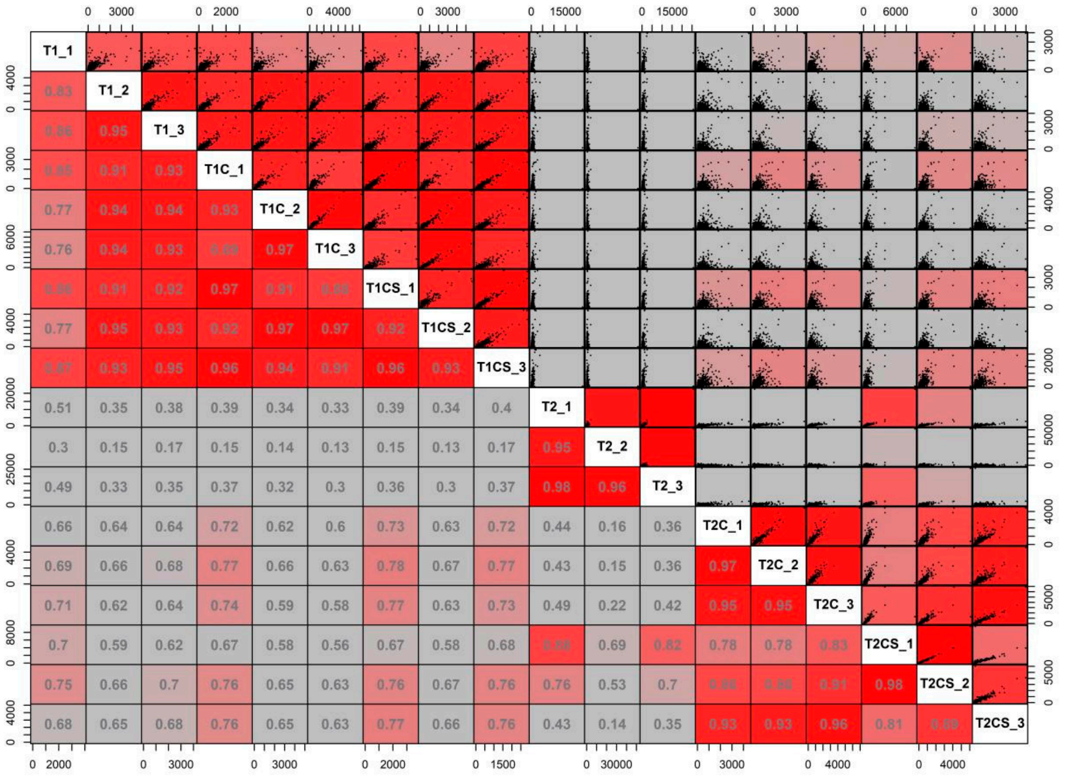

Figure S1 RNA_seq_Assessment. Correlation analysis of 18 samples based on transcriptome data, the number is the correlation coefficient. The closer the number is to 1, the stronger the correlation is. Note: T1 indicates 12 h, T2 represents 30 d, C represents cadmium treatment, S represents selenium treatment, and each treatment has three repetitions.

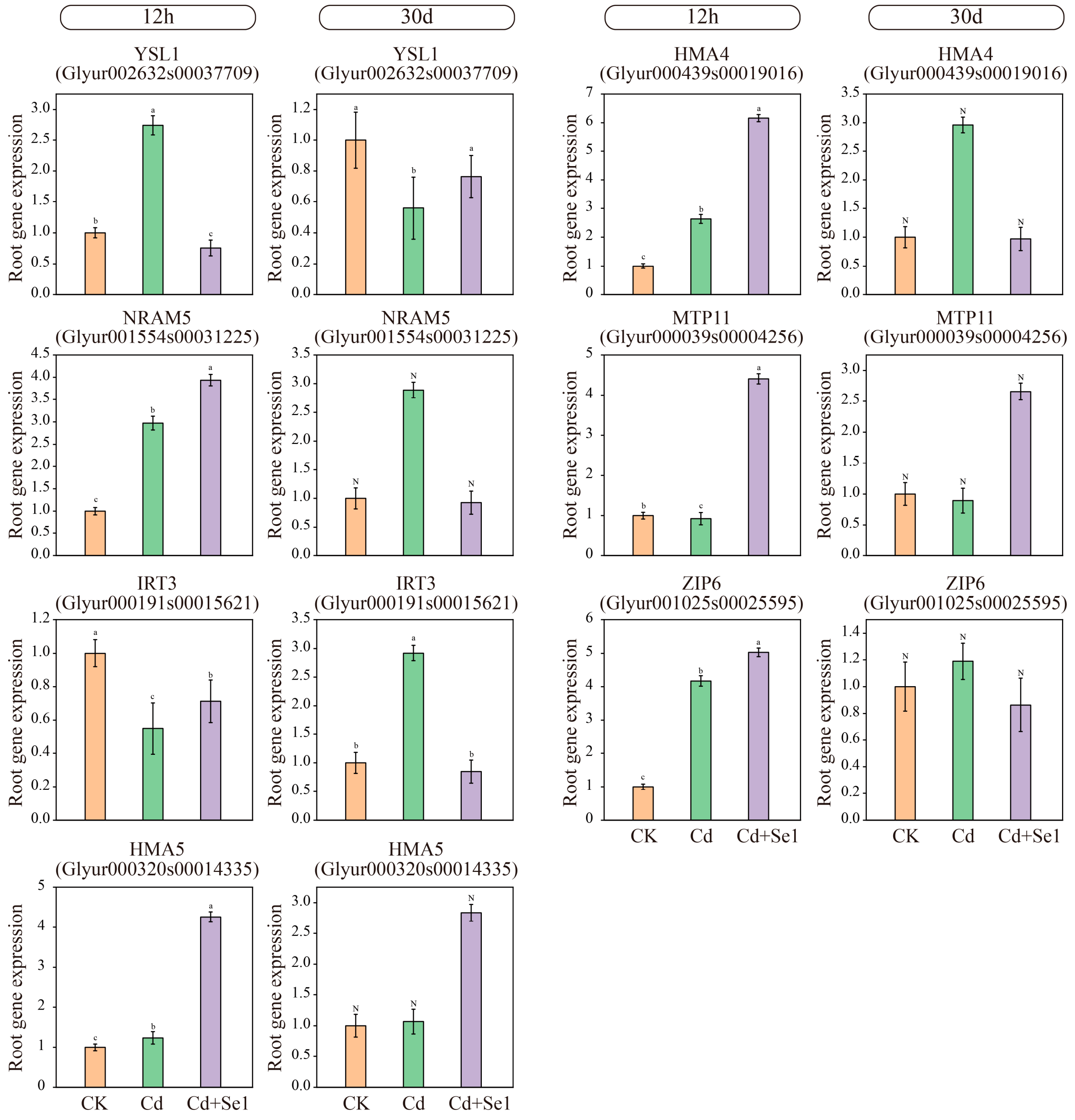

Figure S2 Validation of RNA-Seq results for Cd transporter genes by qRT－PCR. The results were shown as mean ± standard deviation values (n = 3). The small letters indicate significant differences among the different treatment groups ($P < 0.05$). N indicates no significance difference.
